# A Brief Paper on the Effects of Alcohol and Tobacco, on the Human System

**Published:** 1871-05

**Authors:** N. S. Davis

**Affiliations:** Chicago, Illinois


### A BRIEF PAPER ON THE COMPARATIVE EFFECTS OF ALCOHOL AND TOBACCO, ON THE HUMAN SYSTEM.

By N. S. DAVIS, M.D., Chicago, Illinois.

Prepared by the request of the Chicago Medical Society, and read at the Regular Meeting, December 26, 1870.

(*Continued from March No.*)

*Tobacco:*—In regard to the effects of tobacco I will be brief. Sufficient was said concerning the history, botanical relations, and chemical composition of this potent drug, in the paper read by Dr. Emmons at a recent meeting of this Society. The alkaloid and oil, which constitute the principal active ingredients in tobacco, both exert a decided and characteristic influence when introduced into the human system. When given in full doses, either by mouth or rectum, they rapidly reduce the force of the respiratory and circulatory functions, alleviate pain, relax the muscular structures, and generally nauseate the stomach. Hence, before ether and chloroform were introduced into practice as anæsthetics, tobacco was not unfrequently used for the purpose of inducing complete relaxation of the muscular and fibrous tissues, to facilitate the reduction of strangulated hernia; the removal of spasmodic action in "*bilious colic;*" the reduction of dislocated bones, etc.

Under the full effects of the drug, the face is pale; the surface cold and moist; the pulse small, thready, and weak; respiration easy but feeble; the voluntary muscles are relaxed; and the nervous sensibility so impaired that the patient, not only feels no pain, but experiences a feeling of numbness, or, more properly, an absence of feeling.

A lady who had been brought fully under the influence of a tobacco enema to relax the muscular coat of the intestines, expressed the effect upon the nervous sensibility by saying she "hardly felt conscious that she had a body, and in consequence thought she must be dying."

From the experiments which have been performed on animals, and from clinical observation, I am led to regard tobacco as a direct and active nerve sedative. And when the doses are small and the system habituated to its effects, it induces a soothing, tranquilizing feeling, exceedingly fascinating, and which speedily begets a love for the article. Hence, notwithstanding its acrid taste and primary nauseating effects, it has become an article of general use in every civilized country on the globe. It is hardly possible, however, that an agent so decided and active in its influence over the functions of the human system when the latter is not accustomed to its impression, should become altogether harmless by habitual use. And yet so numerous are the influences exerted upon man in civilized society, and so varied are the conditions of every day life, that it is extremely difficult to determine the actual influence, either mental or physical, which is produced by the habitual use of any one article like tobacco. Hence, it is not strange that medical literature should present a confused mixture of facts and opinions of more or less contradictory and perplexing character concerning the effects of the agent under consideration. One class of writers, prominently represented by Drs. Anstie and Hammond, place alcohol and tobacco in the same category, and claim for both the power to act, in small doses, as "*accessory*" or "indirect food," or "food-stimulants;" and in larger doses, as narcotics and poisons. In the former capacity, they are represented as not only harmless to a large majority of individuals, but positively beneficial.

By another class of writers, tobacco is regarded as simply stimulant to the nervous system in small doses, and in larger ones sedative and narcotic.

Still another class regard it as sedative and narcotic, especially to the nervous system, in proportion to the quantity used and the temperament of the individual; and they attribute a long catalogue of evils and diseases to its habitual use.

When its use has been commenced in early youth it is claimed to have diminished growth, impaired mental and physical activity, and weakened the digestive and assimilative functions. Its excessive use in adult life has been represented as causing impaired vision, amaurosis, cardiac weakness, muscular relaxation and tremor, indigestion, irritation of fauces, impotency, mental dulness, etc., etc. Most of the statements by all these classes are expressions of opinion rather than deductions from observed facts.

The assumption that tobacco acts as indirect food, or as a substitute for food when used in moderate quantity, and therefore is either harmless or positively beneficial, affords the only plausible ground on which its habitual use can be defended, and it therefore deserves a careful consideration.

It is not easy to perceive more than two ways in which any agent can act as direct or indirect food. 1st, By furnishing actual material for assimilation. 2d, By retarding the processes of metamorphosis and waste, thereby lessening the necessity for new material.

No one will claim that tobacco constitutes material for digestion and assimilation. The quantity absorbed and taken into the system by any ordinary use is too small to be of the least importance in this way.

That it may retard tissue metamorphosis, as claimed by Dr. Hammond, is probably true to a very limited extent; and only indirectly through its sedative action on the vaso-motor nervous system. It does not, like alcohol, appear to exert a direct influence over that play of vital affinity by which new atoms are appropriated to, and old atoms removed from, the tissues.

Dr. Anstie, himself, attaches no importance to any supposed power to retard tissue change, but simply claims that it enables men to feel less inconvenience from fatigue or hunger while doing a given amount of work on a limited supply of food. All his illustrations go to establish this fact, and this alone. He seems to perceive no difference between rendering a man less *conscious* of hunger and actually feeding him; between removing the *sense* of fatigue and the restoration of nervous and muscular power. Hence it is not strange that he should class opium, tobacco, ether, chloroform, etc., together as food-stimuli or substitutes. But is it not just as absurd to assert that a London pauper is either better fed and clothed simply because he does not feel the sensations of hunger and cold as keenly while rolling a quid of tobacco in his mouth, as he would without it, as it would be to claim that the surgeon's knife did not cut, because his patient did not *feel* it while under the influence of chloroform? That a moderate influence of tobacco will make an individual less conscious of present suffering, whether from hunger, fatigue, pain, or anxiety, and therefore more contented and *do n't-care-a-tive*, is apparent to every careful observer. But is there any proof furnished, from any department or condition of human life, that the use of tobacco enables man to live longer on a given amount of food, to do more work in a given time with less physical impairment, or to incur less sickness during an equal exposure to the causes of disease? In other words, are there any facts or statistics which show that those who have used tobacco have had a longer average duration of life than those who have not in the same community? If so, they are unknown to me. There are, however, many facts tending to an opposite conclusion.

In comparing the effects of tobacco with those of alcohol, we find both exerting a sedative influence over nerve sensibility, but the first spends its force mainly upon the vaso-motor and ganglionic nerves connected with the functions of organic life; while the latter acts more directly on the brain and nerves of animal life. Hence the first, when taken in poisonous doses, destroys life by cardiac paralysis or syncope, and the latter, either by cerebral insensibility or apnœa.

The primary influence of tobacco is limited to the nerve structures, influencing secretion, assimilation, nutrition, and disintegration only by indirection; while alcohol extends its direct action to the properties of all other structures as well as those of the brain, and modifies tissue changes as directly as it does cerebral sensibility.

We find nothing in our reading or clinical observations which sustains the idea that these agents are antagonistic, or that the effects of one antagonize those of the other. On the contrary, we have long been satisfied that the thirst, coupled with the sense of uneasiness in the chest and cardiac region, accompanying the use of tobacco, constituted one of the most active incentives to the use of alcoholic drinks.

And yet, so far is the one from antagonizing the effect of the other, that the use of both undermines the health of the individual more rapidly and certainly than the same amount of either would alone. In regard to the pathological effects produced by the habitual use of tobacco, we will detain you to allude to but three groups of cases that have come under our observation.

The first group present some form of irritation in the lining membrance of the fauces and pharynx, sometimes extending to the mouth. In some cases it consists in follicular hypertrophy; in others, there is more diffusive redness with dryness, especially in the pharynx; and in others, though rarely, there are patches of erythematic redness and tumefaction in the buccal mucous membrance, with a very annoying sense of heat and dryness. We have met with many of these chronic cases of irritation in the mouth and throat, that had resisted every variety of treatment for years, until the use of tobacco was omitted, when they speedily became well. In one instance, a man about 24 years of age, who had suffered severely from the disease in the mouth and throat, tested its connection with the use of tobacco by omitting the use of the latter from six to twelve months at a time, three different times. The disease uniformly disappeared in a few weeks after omitting its use, and as certainly returned in two or three months after resuming it. In a few instances the same follicular irritation has appeared in the stomach.

The second group of cases embrace a series of anomalous, and sometimes very distressing affections of the chest. They vary, in different cases, from a simple feeling of uneasiness, oppression, or weakness in one or both sides of the chest, to that of positive pain, and periods of such extreme exhaustion that the patient is greatly alarmed, and complains of want of breath, sometimes palpitation, and is afraid to lie down at night. It is only a few weeks since that a gentleman called on me from the country, complaining of almost constant uneasiness, and sense of exhaustion in his chest, frequent sighing, what he called *quivering* of the heart and epigastrium, a feeling as though he would lose his breath, and almost entire inability to sleep. These symptoms had been steadily increasing on him for a year, during which time he had used a variety of remedies without relief. His pulse was regular but rather weak, respiration perfectly regular and easy, but rather less full than natural, nutrition fair, and general look of fair health. The most careful and thorough examination disclosed no sign of disease in the heart or lungs or any other internal organ. On inquiry, I found he had been for many years an inveterate user of tobacco, both by smoking and chewing: and since he began to suffer the bad feelings in his chest to such a degree as to interfere with his sleep, he had often walked the floor of his house the greater part of the night with his pipe in his mouth. Suspecting that the tobacco had induced a perverted and depressed condition of the functions of the thoracic ganglia and plexuses of the sympathetic nerve, I persuaded him to abandon at once the further use of the article in any way, and prescribed strychnine in doses of $\frac{1}{32}$ part of a grain before each meal, and 20 grains of hydrate of chloral at bedtime, to be repeated in two hours if sleep was not induced.

He was to exercise moderately in the open air daily, live on plain food, and use no stimulating drink. During the first three or four days he was very uneasy, and complained of such weakness as to keep himself in a constant state of alarm. He persevered, however, and at the end of two weeks he had recovered a fair appetite and strength, slept well with the aid of a very moderate dose of hydrate of chloral at bedtime, and complained but little of the morbid sensations in his chest.

Several years since, another case still more exaggerated came under my care. It was a young man connected with a travelling circus. The amount of tobacco he had consumed, chiefly in the form of cigars, was enormous.

He had become subject to a spasmodic cough, hypertrophy of the follicles of the fauces, and such a sense of sinking and tremulousness in his chest, that he almost feared to walk across his room in the day, or even to try to sleep at night. His countenance was depressed and anxious, his skin cool, and his pulse weak.

He had resorted to a variety of stimulants, tonics, etc., but without relief. Finding no physical signs of disease in his chest, I was at first much puzzled in relation to the nature of the case. A few days observation, however, satisfied me that, aside from the follicular inflammation of the fauces, the whole train of distressing symptoms were dependent on the altered condition of the thoracic and abdominal gaglia of the sympathetic nerve produced by tobacco. Its further use was consequently entirely prohibited, and although he suffered almost every variety of morbid sensation for the first week, yet under a mild tonic and soothing treatment he fully recovered, and has remained well since.

These are exaggerated cases from great excess in the use of the article, but they serve to demonstrate the nature of slighter cases that are presenting themselves almost every day, in which men from all ranks of society complain of just enough pain, or sense of oppression, or feeling of exhaustion in the chest, to make them uneasy lest there might be disease of the lungs or heart; and yet in whom the most careful examination can detect no indications of disease in these organs.

The third group of cases includes those in which the principal symptoms are muscular weakness, sense of unsteadiness in walking, irregular morbid sensations in the extremities, and occasional intermittence or irregularity of pulse. These are symptoms that are identical with those belonging to the early stage of locomotor ataxy. In only two instances have I been able to verify the fact that they were caused by the use of tobacco. Entire omission of the use of tobacco, moderate exercise in the open air, electricity, and small doses of strychnine removed the symptoms in a few weeks.

This brief allusion to the more prominent pathological effects of the habitual use of tobacco, has been made for the purpose of illustrating more fully the fact that its principal action is on the nervous functions of organic life, and that such action is directly sedative. It does not, like alcohol, produce intoxication or anæsthesia of the brain, nor directly interfere with nutrition or disintegration, except in so far as it modifies the vaso-motor nervous action. Consequently its destructive effects on the mental and physical condition of those who use it, bear no comparison to those of alcohol.

That the functions of the human system are capable of adjusting themselves to the effects of a moderate and regular use of both these agents, to such a degree as to maintain health and long life, is abundantly proved by daily observation. That both, however, induce more or less of an unnatural and injurious condition of the physical organization, impairing the activity and perfect healthfulness of its functions, is equally proved by the most obvious facts.

